# Assessment of potential contamination, ecological risk, spatial analysis, and source apportionment of soil heavy metals in a highly industrialized coal–mining region

**DOI:** 10.1007/s10653-026-03099-7

**Published:** 2026-03-02

**Authors:** Ahmet Altin, Bekir Fatih Kahraman, Sinem Çolak, Koray Alper, Süreyya Altin, Ferruh Niyazi Ayoğlu

**Affiliations:** 1 Department of Environmental Engineering, Zonguldak Bülent Ecevit University, 67100 Zonguldak, Turkey; 2 Department of Chemistry and Chemical Processing, Caycuma Vocational School of Food and Agriculture, Zonguldak Bülent Ecevit University, 67900 Caycuma, Zonguldak, Turkey; 3 Department of Public Health, Faculty of Medicine, Zonguldak Bülent Ecevit University, 67100 Zonguldak, Turkey

**Keywords:** Zonguldak (Turkey), Positive matrix factorization (PMF), Ecological risk assessment, Soil pollution, Pollution load index (PLI), Enrichment factor (EF)

## Abstract

Assessing soil quality in industrial coal-mining regions is critical for ensuring environmental sustainability and public health. This study provides a comprehensive assessment of heavy metal (HM) contamination, ecological risk, and source apportionment in the Zonguldak province of Turkey. By utilizing systematic soil sampling, the research integrates statistical evaluation and spatial mapping with multiple contamination indices, including the Enrichment Factor (EF), Geo-accumulation Index (I_geo_), Pollution Load Index (PLI), and Potential Ecological Risk Index (PERI). Results revealed that mean concentrations of Co, Cu, Hg, Cd, Zn, and Ni significantly exceeded crustal and global soil averages. Furthermore, high coefficients of variation for elements such as Cu and Zn suggested significant anthropogenic influences. Spatial analysis, supported by I_geo_ and EF indices, identified critical pollution hotspots near the urban centers of Ereğli, Alaplı, and Zonguldak, as well as the Gülüç River basin. The overall ecological assessment indicated moderate ecological risk (PERI) across most of the study area, with Hg posing the highest individual risk based on average Er values. PMF analysis identified five potential sources contributing to heavy metal enrichment: coal mining and coal-based emissions, geogenic origins, industrial processes, agricultural activities, and mixed sources. This integrated approach establishes a robust scientific baseline for the effective management of heavy metal pollution in coal-dependent industrial landscapes.

**Supplementary Information:**

The online version contains supplementary material available at 10.1007/s10653-026-03099-7.

## Introduction

One of the negative consequences of increasing industrialization is the evident worsening of the soil heavy metal (HM) pollution (Duan et al., 2023; Khoshyomn et al., 2024). Among the most significant soil pollutants of both natural and anthropogenic origin, HMs pose a serious problem due to their elevated toxicity, high mobility and resistance to degradation (Fan et al., 2023). They can be easily transported to the tissues of living organisms including humans through the food chain (Goni et al., 2025) causing serious and irreversible health problems (Sharma et al., 2018; Wu et al., 2018; Zhou et al., 2019). In addition, heavy metals can disrupt soil ecosystems. They can reduce the number of biological organisms in the soil and have toxic effects on plants, thus causing reduced agricultural productivity (Huang et al., 2019; Zhan Liu et al., 2023).

Mining activities, fossil fuels, metal processing plants, and industrial wastes are significant anthropogenic sources of heavy metals in soil (Fry et al., 2020; Khan et al., 2021). Coal mining is responsible for the release of significant amounts of heavy metals into the environment and thus causes serious health problems (Cheng et al., 2020; Li et al., 2014; Sun et al., 2019). Coal used in thermal power plants for energy production can increase the levels of heavy metals in soil through various geochemical pathways. Industrial activities are another primary source of heavy metals in soil. It is generally observed that the soil metal content increases in areas with intense industrial activities. Heavy metal pollution can be observed in the soil, especially in areas where industrial facilities smelt non-ferrous metals (Xu et al., 2021). Heavy metals such as lead, zinc, and cadmium emitted from smelting facilities have been shown to account for 40–73% of anthropogenic emissions (Xu et al., 2021). Metal accumulation in these regions is several times greater than in areas far from anthropogenic sources. However, due to the long-range atmospheric transport, high metal concentrations can also be detected in areas far from industrial areas (Yaylalı-Abanuz, 2011).

There are numerous factors and stressors that create a synergistic effect on pollution patterns and risks. For example, it has been shown that, in regions hosting mining activities, the spatial distribution and transport of elements such as Cd, Pb, Hg, and As in the soil vary under different land use conditions (such as forestland, farmland, grassland, and bareland), and that the prevailing wind direction in semi-arid regions is also a determining factor (X. Zhang et al., 2024). The interactions between industrial emissions, mining activities, soil properties (such as pH and soil type), and land use might increase heavy metal accumulation and mobility beyond the impact of each factor individually (Zhaoyue Liu et al., 2022). These and similar studies demonstrate that climate change-related factors and changes in land use, together with industrial emissions, have complex synergistic effects that increase or modify heavy metal contamination in soil, and that multi-factor risk assessment and management approaches tailored to the dynamic interplay of environmental stressors are needed in coal mining areas. Therefore, studies on the assessment and management of HM pollution in soils surrounding coal mines and related industries are crucial (Zhu et al., 2024).

Although the literature on soil heavy metal contamination associated with coal mining and coal combustion activities has expanded significantly since 2020, the geographical distribution remains markedly uneven, with studies concentrated primarily in China, India, Bangladesh, and other Asian countries (Rouhani et al., 2023). Despite the limited literature on Turkey, recent studies in active coal basins, including Kütahya–Tunçbilek, Kahramanmaraş–Afşin-Elbistan, Çanakkale–Biga, and Sivas–Kangal, have partially altered this perception (Akbay et al., 2022; Ateş et al., 2022; Esen et al., 2021; Parlak et al., 2024). These studies show that in Turkey, radionuclides such as ^226^Ra and ^232^Th, as well as heavy metals such as As, Ni, Cr, and Co, exceed background levels in coal, fly ash, and surrounding soils, creating significant enrichment and resulting in lifetime cancer risk (ELCR) that is high in some regions compared to international standards (Akbay et al., 2022; Ateş et al., 2022; Esen et al., 2021). In ecological risk assessments, Uranium (U) in Afşin-Elbistan and Nickel (Ni) in Tunçbilek stand out as medium-level risk factors, while human health risk analyses reveal that carcinogenic risks from Cr and Pb in the Çanakkale–Biga example may exceed acceptable thresholds (Akbay et al., 2022; Parlak et al., 2024). Source apportionment approaches confirm that heavy metal pollution is not solely attributable to mining; it is fed by multiple sources such as thermal power plant emissions, traffic, and agricultural inputs (Akbay et al., 2022; Ateş et al., 2022; Parlak et al., 2024). However, the lack of a current, integrated, and source-focused soil contamination study for the Zonguldak Basin, one of Turkey’s most established and intensive coal production regions, represents a significant and unaddressed research gap in the literature.

To effectively manage and mitigate heavy metal pollution, understanding their spatial distribution, accurately assessing associated risks, and conducting source apportionment are critical (Kowalska et al., 2018; Su et al., 2022; Wang et al., 2024). Spatial analysis techniques, such as Geographic Information Systems (GIS) and geostatistical methods (e.g., kriging, inverse distance weighting), are widely employed to map heavy metal concentrations and identify pollution hotspots (El-Sorogy et al., 2025; Fei et al., 2020; Kalinović et al., 2024; Zhang & Zhang, 2021). Furthermore, various indices are used to assess the degree of soil contamination and potential ecological and human health risks. These include pollution indices like the Enrichment Factor (EF) and Geo-accumulation Index (Igeo), ecological risk indices such as the Potential Ecological Risk Index (RI/PERI), and other single–factor indices, like the Pollution Load Index (PLI) (Altin, 2023; Weissmannová & Pavlovský, 2017). As for source apportionment, the PMF model is widely used in studies using water, soil, particulate matter, and sediment samples to identify potential pollution sources (Chen et al., 2013; Liang et al., 2017; Tan et al., 2016; Zhu et al., 2024). Unlike traditional methods, it eliminates the negative value problem and utilizes sample–specific data uncertainty estimates (Brown et al., 2015; Liu et al., 2024; Song et al., 2018). While these aforementioned methods have been used widely, integrated assessments combining all these approaches for management of high–risk regions in which multiple factors are synergistically at play are scarce. As a major coal mining region and host to several industrial facilities, Zonguldak Province (Turkey) represents a critical example for such an integrated assessment.

It is hypothesized that the heavy metal distribution in the study area, due to the intense mining, heavy industrial activities, and other environmental factors in the region, has exceeded the local geogenic background levels and created significant environmental pressure. In this context, the objectives of this study are to (1) Lay out a general overview of the heavy metal levels in Zonguldak soils and their interrelationships using statistical methods (2) Map the spatial distributions of soil heavy metals throughout the study area (3) Quantify contamination levels and ecological risks using various indices (EF, I_geo_, PLI, and PERI) (4) Identify and classify HM pollution sources by using the PMF model. This integrated approach will provide a scientific basis for the assessment and effective management of metal pollution in the soil environment within Zonguldak Province.

## Materials and methods

### Study area

**Fig. 1:**
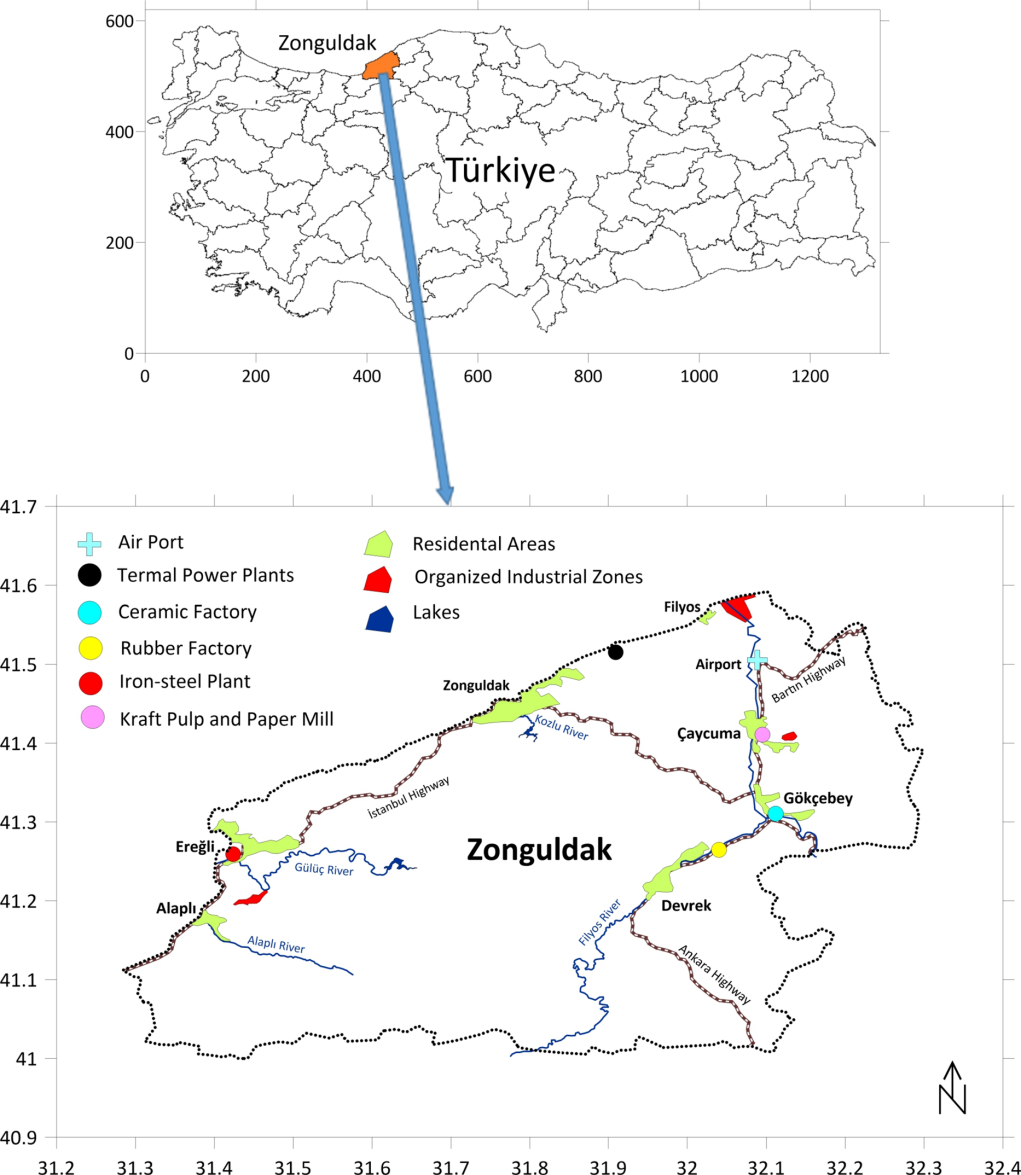
Map of the sample collection area

Zonguldak Province (41° 27′ 23″ N 31° 47′ 55″ E) is one of the 81 administrative provinces of Turkey. It is located on the northern coast of the country along the Black Sea with a surface area of 3294.9 km^2^ (Fig. 1) (Bayrakli, 2023). Characterized by its mountainous terrain, Zonguldak is largely covered by forests and possesses a lot of rivers (Keskin Citiroglu et al., 2017). The Province of Zonguldak is characterized by a humid subtropical climate, experiencing an average annual temperature of 12.4 °C and receiving approximately 915 mm of precipitation annually (Aksoy, 2024). Coal mining, energy production, and metallurgy are the biggest industrial activities in the area (Öztürk Pulatoğlu, 2024; Zeydan & Öztürk, 2021). In addition to large-scale Kraft paper, ceramics, and rubber production facilities, there are also three organised industrial zones where production is carried out in different sectors, including iron and steel rolling, aluminium production, textiles, metal recovery from waste slag, and natural gas processing. These activities, along with other potential sources like residential heating via coal, agricultural practices, and vehicle emissions, have raised concerns about soil pollution in Zonguldak (Batur, 2022).

**Fig. 2:**
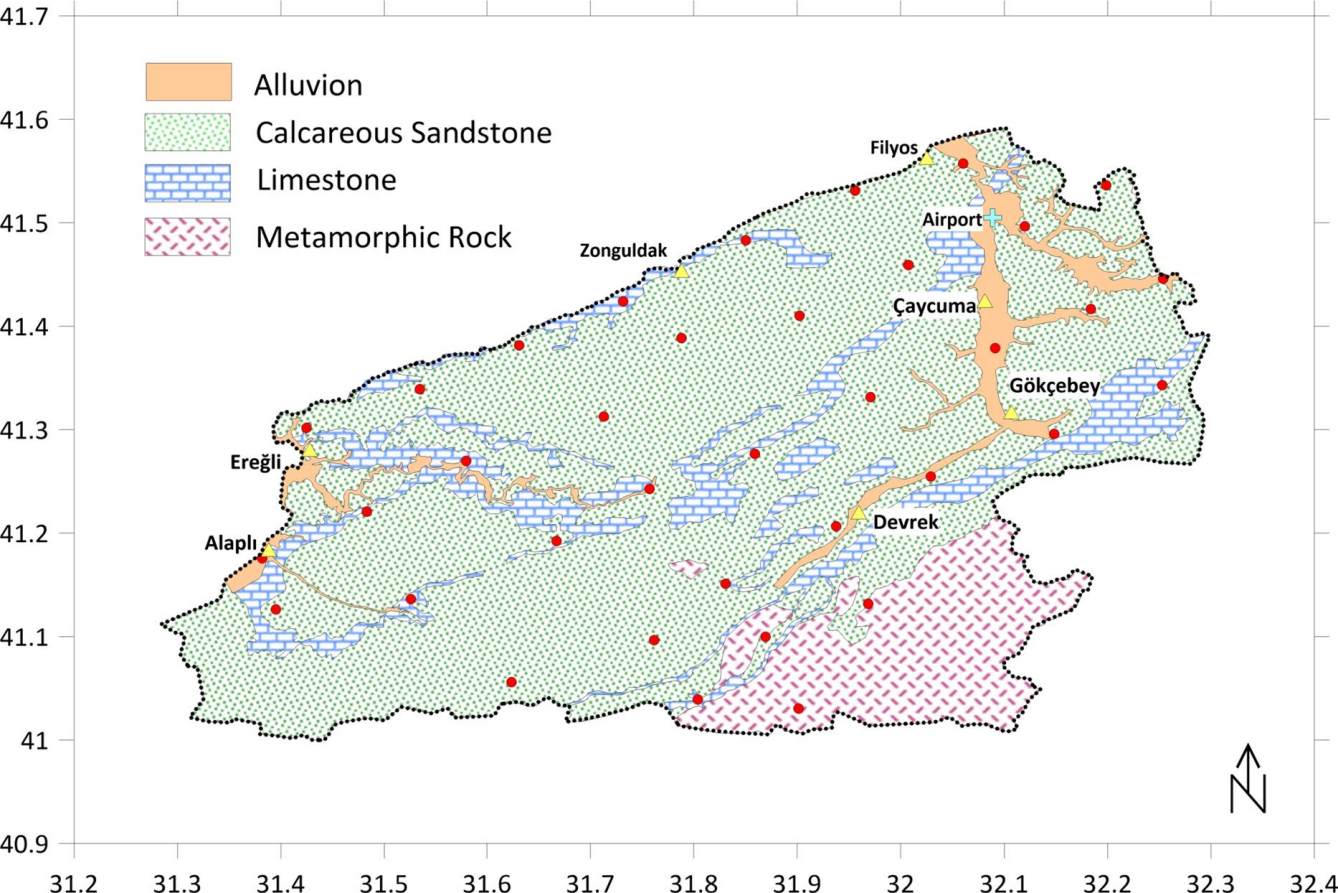
Sample points and simplified geological map

A simplified geological map of the study area and sample points is presented in Fig. 2. Zonguldak, located in the western part of the North Anatolian Mountains, which were shaped by the Alpine Orogeny (Alpine mountain-building movements), is in a tectonically active region due to its proximity to the North Anatolian Fault. The region contains Paleozoic rocks (Devonian Yılanlı Formation), Carboniferous sedimentary rocks (sandstone, shale, and coal seams), Mesozoic and Cenozoic rocks (especially Cretaceous limestone, which is abundant in caves, and volcanic rocks, although limited in extent) (Akkaya & Keskin Çıtıroğlu, 2012; Bacak & Yılmazer, 2011). Sandy-clayey horizons are common in the Eocene basin around Çaycuma (Kahraman et al., 2017). Limestones occur as thin intercalations rather than distinct horizons. The steep slopes, particularly in areas formed by metamorphic rocks and granites, are highly competent and stable. Figure 2 shows that limestone outcrops are seen in the north, inland, and western regions. However, the steep topography to the south-southeast contains metamorphic rocks. Alluvial plains have developed in the valley beds due to the influence of the Filyos and Gülüç rivers.

### Sampling strategy and sample pre-treatment

For a province-wide sampling initiative, 36 sampling locations (Fig. 2) were strategically selected to ensure a homogenous representation of the entire province. These locations were distributed to allocate approximately 100 km^2^ of coverage per point, and the coordinates of the sampling points were given in Table S1. Soil samples were extracted from a depth of 0–30 cm, with each sample comprising a composite of three sub-samples collected within a 4 m^2^ radius around each designated point. The collected soil samples were subsequently transported to the laboratory for analysis. Upon arrival, the samples underwent air-drying at room temperature to eliminate moisture. Following the drying process, the samples were crushed using a mortar and pestle to disaggregate any large clumps and remove extraneous materials, such as stones, roots, or debris. The crushed samples were then subjected to sieving through a 10-mesh sieve to achieve a consistent particle size. Approximately 100 g of each soil sample were oven-dried at 40 °C for 24 h and subsequently stored in sealed bags for additional processing.

### Heavy metal analysis procedure

A modified version of the USEPA3051A method was used for soil heavy metal extraction. Briefly, 200 mg of soil samples were treated with an extraction solution including 5 ml HNO_3_, 3 ml HCl, and 1 ml H_2_O_2_ in a microwave oven (Berghof Speedwave). The temperature program of the microwave digestion procedure is presented in Table S2. Heavy metal analyses were performed with ICP-MS (Nexion, 300D, PerkinElmer, Inc. Waltham, MA, USA) coupled to a CETAC ASX 520 autosampler. The instrumental operating conditions were set as: RF power was 1600 W, Argon gas flow and carrier gas (inner) flow rates were 17 L min^−1^ and 1.3 L min^−1^, and sample intake rate was 0.5 mL min^−1^. All experiments were performed in standard mode with an integration time of 0.3 s and 3 sample repetitions. The calibration range was prepared individually for each metal from a 1000 mg L^−1^ standard solution. A six–point calibration (0.01, 0.025, 0.05, 0.1, 0.2, and 0.25 mg.L^−1^) was used for each metal. Recovery performance of the procedure was tested on a certified reference material SQC001-30G (Lot: LRAB7490 Sigma-Aldrich, USA). The average recovery values of elements were in the range of 62.3–110.4%. The details of the analyses (detection limit (LOD), limit of quantification (LOQ), and recovery percentages for 17 elements) are provided in Table S3.

### Methodological approaches for contamination assessment and source apportionment

After a preliminary evaluation with descriptive statistics, a correlation analysis was conducted on the heavy metal data. Pearson correlation analysis was performed between all 17 elements to determine the multi-element relationships in soil samples. A hierarchical clustering technique was then applied to all element concentrations (variables)—which were quantitatively analyzed and are thought to be influential in differentiating the soil samples—in order to identify the groups they form. In the study, the group average was used as the method, and Euclidean as the distance type. In order to show the differences and similarities between the metals in the soil samples, a dendrogram was obtained. Origin PRO 2018 software was used to calculate descriptive statistics, correlation, and Hierarchical Cluster Analysis (HCA) parameters.

Spatial distribution analysis maps and visualizes the varying concentrations of heavy metals in soil using different interpolation techniques (Liu et al., 2024). Its purpose is to identify pollution hotspots and understand the spatial patterns of heavy metals to link them to potential sources, aiding in the development of targeted pollution control measures and informed soil management strategies (Su et al., 2022). The Surfer 15 software was used to draw spatial distribution maps and other maps. The spatial distribution maps were obtained using the ordinary kriging method.

Several contamination and ecological indices were adopted in this study for the evaluation of the analysis results:

*Geoaccumulation index (Igeo)* The Geoaccumulation Index (Igeo) is a widely utilised quantitative indicator for assessing the intensity of pollution by individual heavy metals in soil (Kowalska et al., 2018).

1 $${\mathrm{I}}_{{{\mathrm{geo}}}} {\text{ = log}}_{{2}} \left[ {\frac{{{\mathrm{C}}_{{\mathrm{n}}} }}{{{1}{\mathrm{.5B}}}}} \right]$$

where C_n_ is the concentration value and B is the background element concentration. Values of Igeo help classify soil into different pollution levels, from uncontaminated to extremely contaminated (Kowalska et al., 2018). In this study, the background element concentrations used to calculate the Igeo value are the 10th percentile values of the dataset.

*Enrichment factor (EF)* The Enrichment Factor (EF) is a measure primarily used to quantify anthropogenic influences on heavy metal pollution in soil. It evaluates the extent of heavy metal enrichment by normalising the concentration of a target metal against a reference element that exhibits low variability in its natural occurrence, such as Fe, Mn, Al, Ca, or Ti (Weissmannová & Pavlovský, 2017).

2 $${\text{EF = }}\frac{{\frac{{{\mathrm{C}}_{{\mathrm{n}}} }}{{{\mathrm{C}}_{{\mathrm{r}}} }}}}{{\frac{{{\mathrm{B}}_{{\mathrm{n}}} }}{{{\mathrm{B}}_{{\mathrm{r}}} }}}}$$

where C_n_ is the target element concentration in the sample, C_r_ is the reference element concentration in the sample, B_n_ is the background concentration of the target element, and B_r_ is the reference element background concentration. In this study, Fe is chosen as the reference element due to its relatively consistent and stable results.

*Ecological risk ındex (Er)* The Ecological Risk Index (Er), often referred to as the single ecological risk factor, is a component of the potential ecological risk assessment that quantifies the ecological risk posed by an individual heavy metal. It accounts for the inherent toxicity level of the heavy metal and the contamination factor of that metal, reflecting the sensitivity of the environment to the specific heavy metal pollution (Wang et al., 2024; Weissmannová & Pavlovský, 2017).

3 $${\mathrm{E}}_{{\mathrm{r}}}^{{\mathrm{i}}} {\text{ = T}}_{{\mathrm{r}}}^{{\mathrm{i}}} \times {\mathrm{C}}_{{\mathrm{f}}}^{{\mathrm{i}}}$$

4 $${\mathrm{C}}_{{\mathrm{f}}}^{{\mathrm{i}}} { = }\frac{{{\mathrm{C}}_{{\mathrm{m}}} }}{{{\mathrm{C}}_{{\mathrm{r}}} }}$$

where ${\mathrm{T}}_{{\mathrm{r}}}^{{\mathrm{i}}}$ is the toxicity response coefficient of the element, ${\mathrm{C}}_{{\mathrm{f}}}^{{\mathrm{i}}}$ is the contamination factor, C_m_ is the average concentration of the target element, and C_r_ is the pre-industrial background concentration of the target element. ${\mathrm{T}}_{{\mathrm{r}}}^{{\mathrm{i}}}$ used in this study are 40, 30, 10, 5, 5, 5, 5, 2, and 1 for Hg, Cd, As, Co, Cu, Pb, Ni, Cr, and Zn, respectively.

*Potential ecological risk Index (PERI)* The Potential Ecological Risk Index (PERI), also widely known as the Risk Index (RI) or Hakanson Risk Index (HRI), provides a comprehensive assessment of the potential ecological risks posed by multiple heavy metals in environmental matrices like soil or sediment. It integrates the concentrations and toxicity response factors of individual heavy metals to quantify their combined ecological hazard, offering a method to evaluate the potential degree of harm to the ecosystem (Kowalska et al., 2018; Su et al., 2022).

5 $${\text{PERI = }}\mathop \sum \limits_{{\text{i = 1}}}^{{\mathrm{n}}} {\mathrm{E}}_{{\mathrm{r}}}^{{\mathrm{i}}}$$

*Pollution load ındex (PLI)* The Pollution Load Index (PLI) is a comprehensive index used to characterise the overall degree of heavy metal pollution at a given site or area.

6 $$PLI = \sqrt[n]{{CF_{1} CF_{2} \ldots CF_{n} }}$$

where CF is the contamination factor. A PLI value greater than 1 indicates that the soil is polluted, while a value of 1 suggests pollution levels are similar to background levels, and less than 1 indicates no pollution. PLI offers a simple and comparative means for assessing environmental quality (Hossain Bhuiyan et al., 2021; Kowalska et al., 2018).

### Positive matrix factorization (PMF) model

The PMF model is a powerful multivariate receptor modelling tool extensively employed for source apportionment in environmental studies. PMF offers significant advantages over methods such as PCA and factor analysis, facilitating the understanding of the resulting factors, being quantitative, and being able to use even data sets with large gaps. The model equation is presented below (Fei et al., 2020; Singh et al., 2023):

7 $${\mathrm{X}}_{{{\mathrm{ij}}}} { = }\mathop \sum \limits_{{\text{k = 1}}}^{{\mathrm{p}}} {\mathrm{g}}_{{{\mathrm{ik}}}} {\mathrm{f}}_{{{\mathrm{kj}}}} {\text{ + e}}_{{{\mathrm{ij}}}}$$

where X_ij_ denotes jth pollutant concentration on ith sampling point, p is the factor number, g_ik_ represents the contribution of source kth to ith sampling point, f_kj_ is the pollutant concentration of jth pollutant from kth source, and e_ij_ is the residual matrix. The value of the objective function Q is minimized via the following equation (Fei et al., 2020; Singh et al., 2023):

8 $${\text{Q = }}\mathop \sum \limits_{{\text{i = 1}}}^{{\mathrm{n}}} \mathop \sum \limits_{{\text{j = 1}}}^{{\mathrm{m}}} \left( {\frac{{{\mathrm{e}}_{{{\mathrm{ij}}}} }}{{{\mathrm{u}}_{{{\mathrm{ij}}}} }}} \right)^{{2}}$$

where u_ij_ denotes the uncertainty value. When pollutant concentration is higher than the method detection limit (MDL), the following equation is used for uncertainty determination (Guan et al., 2018):

9 $${\mathrm{u}}_{{{\mathrm{ij}}}} { = }\sqrt {\left( {{\mathrm{e}} \times {\mathrm{c}}} \right)^{{2}} {\text{ + MDL}}^{{2}} }$$

where e is the error fraction, c is the pollutant concentration, and MDL is the method detection limit. Otherwise (Guan et al., 2018):

10 $${\mathrm{u}}_{{{\mathrm{ij}}}} { = 5/6} \times {\mathrm{MDL}}$$

The source analysis of heavy metals was conducted with EPA PMF 5.0. Heavy metals (Cr, Pb, Hg, As, Zn, Cd, Ni, Cu) were selected after considering the results of the statistical analyses, spatial distributions, and indices. The selection of these heavy metals was justified in detail in section "Source apportionment (PMF analysis)" Source apportionment (PMF analysis). The number of factors was investigated in a range of 4–6 and determined based on the regional investigation of potential sources and model performance parameters. Three samples with outlier data of Hg, Pb, and Cr were not included in the analysis to improve model performance. This modification increased the R^2^ values and improved the distribution of residuals without causing significant changes in factor profiles. Potential outliers were initially identified using robust Mahalanobis distance based on the multivariate metal concentration matrix. Visual diagnostics were performed using box-plots subsequently to assess the extremeness and influence of these observations. Only samples consistently identified as extreme by both statistical and visual criteria were excluded resulting in a conservative outlier removal strategy. Signal-to-noise ratios of the heavy metals participated in the analysis were in a range of 3.7–9, which indicates that variability is not by chance and PMF analysis can be used (Liu et al., 2024). The number of operations was set to 20, the seed number was randomly determined, and the minimum Q value was chosen.

## Results and discussion

### Descriptive statistics

The statistical data from the metal analysis of 36 samples, collected from various locations throughout Zonguldak Province, are presented in Table 1. Standard deviations are considerably high for most of the heavy metals. Coefficient of variation (CV) is a measure of dispersion and variability as well as it might indicate that anthropogenic factors are in effect (Liu et al., 2024; Zhang & Zhang, 2021) and generally around 0.5 is considered a threshold for high variability (Song et al., 2018; Wang et al., 2024) In this study, the elements with high variability were Cu > Sr > Zn > Mo > Mn > Sn > As > Sb > Ba according to CV results. Considering that other elements also have moderate CV values and high standard deviations, including Hg, Pb, and Ni (0.46, 0.43, and 0.43, respectively), anthropogenic effects emerged as a plausible factor meriting further investigation.

**Table 1:** Descriptive statistics of element concentrations in soil samples from Zonguldak (ppm)

	Minimum	Mean	Maximum	SD	CV	MAC
Fe	22,621.06	41,152.46	90,637.27	13,160.08	0.32	
Sr	19.09	131.50	493.01	130.32	0.99	
Mn	176.78	999.31	4723.03	748.08	0.75	
Ba	65.57	286.67	829.55	167.48	0.58	
Co	8.28	21.55	48.18	8.01	0.37	20–50
Cu	1.54	51.14	432.38	77.75	1.52	6–150
Hg	0.14	0.25	0.72	0.11	0.46	0.5–5
Mo	0.29	1.07	4.21	0.81	0.76	4–10
Pb	15.97	30.68	93.92	13.08	0.43	20–300
Sb	0.14	0.38	1.14	0.22	0.58	10
Cd	0.41	0.67	1.29	0.15	0.23	1–5
Sn	1.34	2.54	11.85	1.75	0.69	
Zn	33.95	101.62	582.07	91.97	0.90	100–300
As	0.27	2.23	6.78	1.42	0.64	15–20
Cr	26.86	61.38	114.52	20.57	0.34	50–200
V	67.64	121.22	222.03	32.60	0.27	150
Ni	14.79	53.66	113.30	22.86	0.43	20–60

*SD standard deviation, *CV* coefficient of variation, *MAC* maximum allowable concentration for trace metals in agricultural soils (Kabata-Pendias, 2011)

It is important to take into account the geological characteristics of the region (Fig. 2) to interpret the results because heavy metal concentrations can greatly vary with local geological structure. Based on average values, elevated concentrations were noticed relative to the upper continental crust levels (Table S4) for Fe, Mn, Co, Cu, Hg, Pb, Cd, Zn, Cr, V, and Ni, while Sr and Ba concentrations were lower. Considering the dominant calcareous sandstone formation in the region and the presence of limestone intercalations, these high concentrations were expected to be diluted. Areas with metamorphic and/or granitic formations possibly influenced high Fe, Mn, Cu, Zn, Cr, V, and Ni levels (Mimba et al., 2018). Mean concentrations of elements Co, Cu, Hg, Cd, Zn, and Ni were higher than all of the reference values in Table S4, including upper continental crust, world soil average, and European soil average. Therefore, although the geogenic factors have an effect on these results to some degree, there are also signs of anthropogenic effects, especially on environmentally significant heavy metals. Among these heavy metals, mean values of Co, Cu, Pb, Zn, Cr, and Ni are within the range of maximum allowable concentration (MAC) levels (Kabata-Pendias, 2011) proposed for agricultural soils (Table 1); however, surpassing the lower limit is an indication of possible exceedance of natural background levels.

Average concentration values were also compared with the results of studies conducted in the same province (Zonguldak), same country (Turkey), and regions with the same characteristics in terms of coal mining and industrial activities (Table S4). A recent study has focused on a specific district (340 km^2^) in Zonguldak in which hazelnut cultivation is a common agricultural activity and reported relatively lower concentration levels for especially Pb, Zn, Cr, Cu, Ni, and Co (Bayrakli, 2023). This study aimed to reveal a comprehensive picture of the entire Zonguldak Province, which has a surface area of 3294.9 km^2^. This suggests that the general findings for such a large area might not represent specific locations in the area with different characteristics. For the majority of the elements, average concentrations were lower than or comparable to the reports from other studies conducted in different parts of the world. Thus, the results represent the characteristics of an industrialized region.

### Correlation analysis

**Fig. 3:**
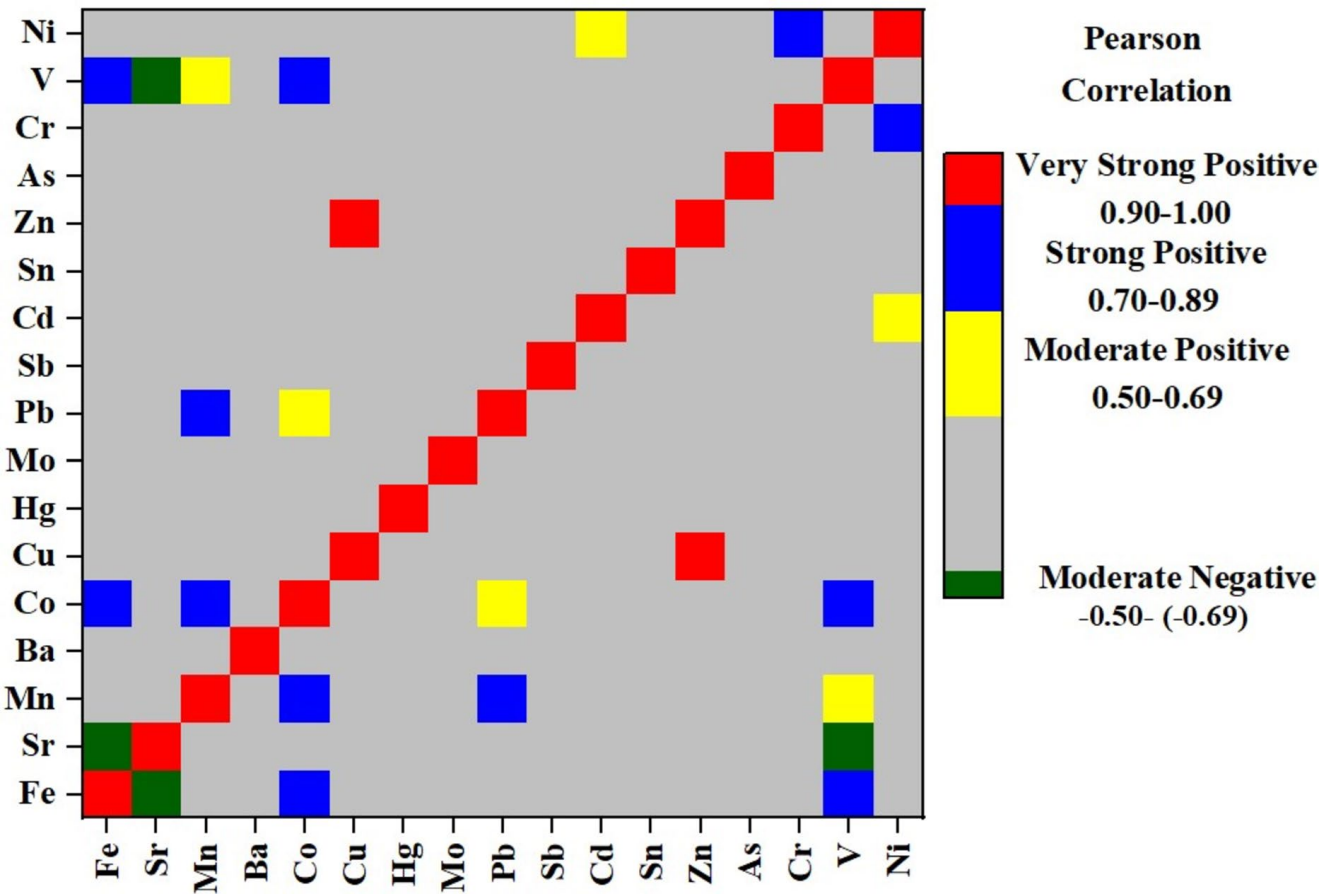
Correlation heat map of metals in soil

The heat map of the correlation analysis is given in Fig. 3. Heavy metals are generally closely correlated with each other. The highest positive correlations were found between Cu–Zn (r = 0.95), Cr–Ni (r = 0.84), V–Fe (r = 0.78), Mn–Pb (r = 0.78), Mn–Co (r = 0.75), Fe–Co (r = 0.75), Co–V (r = 0.72), and V–Mn (r = 0.61) (*p* < 0.01). These high coefficient values show that it is highly likely that these metal pairs have common geochemical behaviours or contamination sources, while other metal pairs with low or negative coefficient values indicate different sources or natural origins (Linnik et al., 2022). The strong correlations of Fe, Mn, and Cr might be linked to lithogenic sources. Cu, Zn, Pb, and Cd correlations have been observed in contaminated soils (Xiaoqian et al., 2023).

### Hierarchical cluster analysis (HCA)

**Fig. 4:**
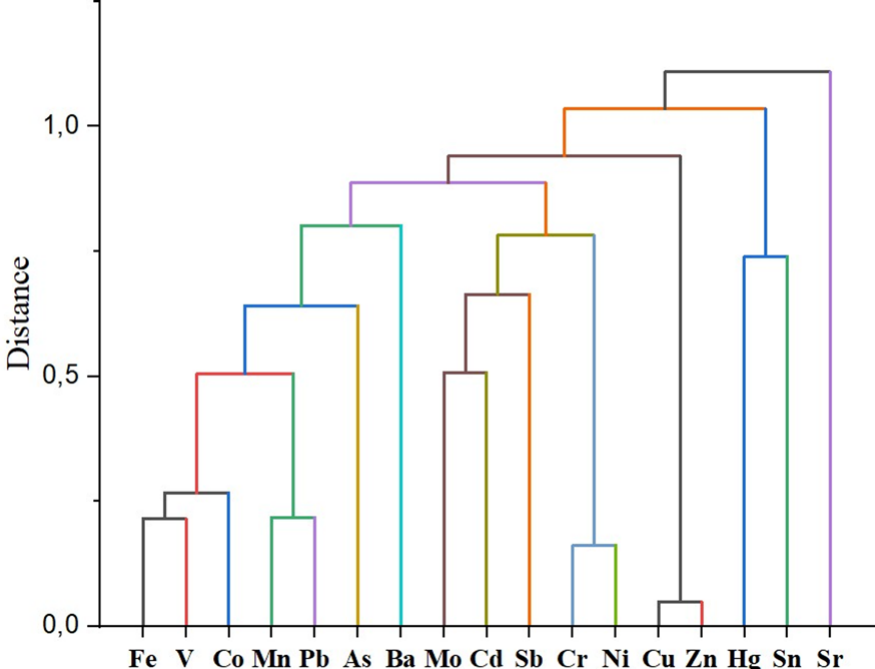
Hierarchical clustering analysis dendrogram of soil samples

The HCA dendrogram presented in Fig. 4 depicts the grouping of heavy metals based on their similarity in distribution patterns across soil samples from Zonguldak Province. In the dendrogram, there are 3 main clusters, and under those clusters, there are clusters of twos and threes with similar behaviors. It is seen that Sr is a separate cluster on its own and is independent of other metals. Sr–Ca substitution is highly expected in minerals (especially in carbonates) because Sr has an ionic radius which is quite resembling that of Ca (Kabata-Pendias, 2011). This can lead to Sr enrichment in carbonate–containing formations like limestones and calcareous sandstones, which are abundant in the study area (Stanienda-Pilecki et al., 2024). It is also essential to consider atmospheric deposition from marine aerosols as an important secondary source for Sr accumulation in terrestrial surface soils. Studies focusing on element distribution in organic surface soils have found that the atmospheric deposition of marine aerosols contributes significantly to Sr levels (Nygård et al., 2012). This influence is often manifested in coastal regions where the intensity of winter storms facilitates the transport of marine aerosols inland (Nygård et al., 2012). The study area, Zonguldak, is located on the northern coast along the Black Sea, making the potential for atmospheric transfer of ions from the marine environment a relevant factor influencing the trace element distribution in the soil. Two elements with high affinity for sulfur and organic matter (Kabata-Pendias, 2011), Hg and Sn, were in the second cluster. In the third large cluster, the other metals formed different small clusters. The large cluster includes Fe, V, Co, Mn, Pb, As, and Ba, most of them having strong correlation coefficients in the range of 0.61–0.78 (*p* < 0.01). Mo–Cd–Sb–Cr–Ni is the second big group, while Cu–Zn couple differentiates from the other two big clusters.

### Spatial analysis of heavy metals

**Fig. 5:**
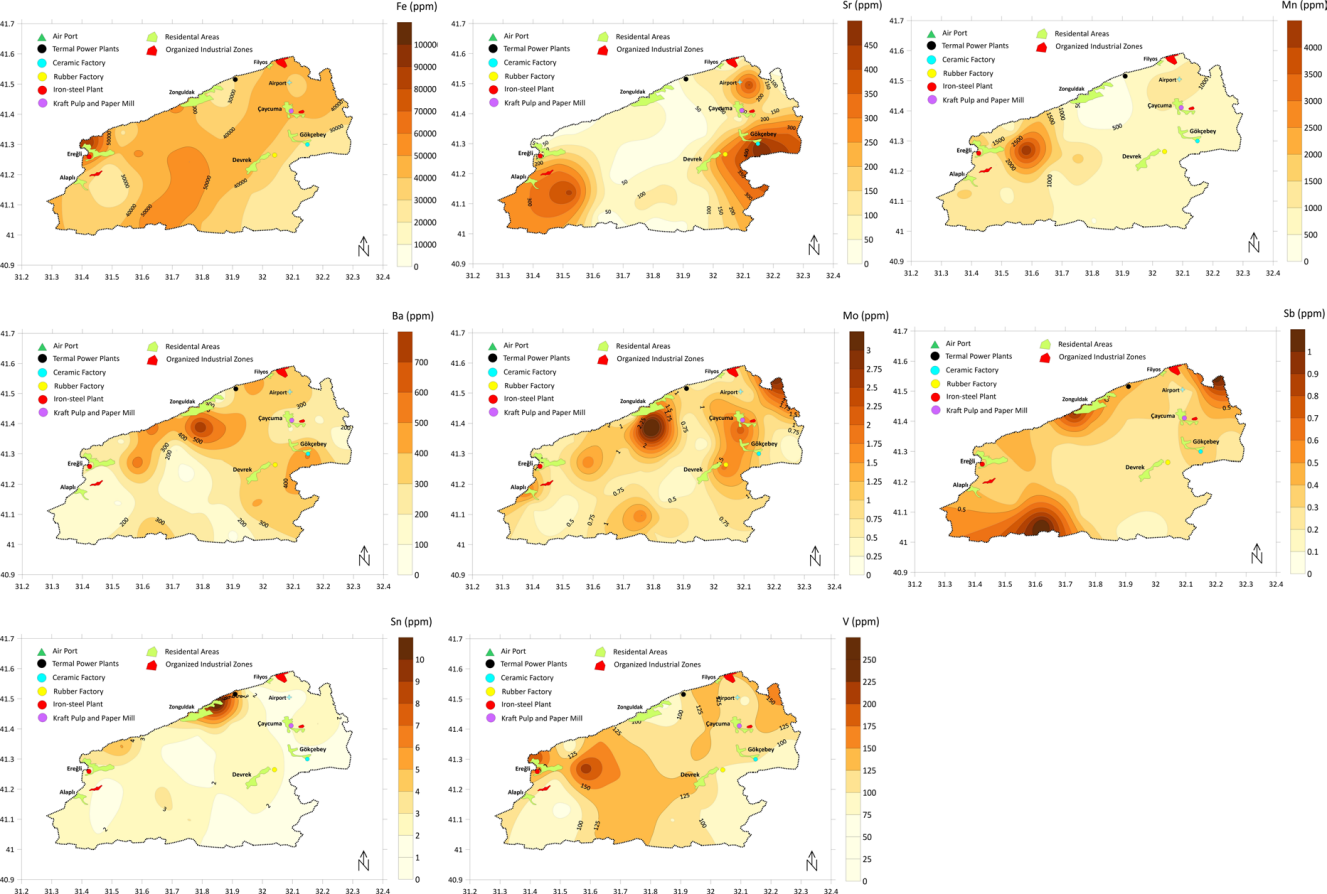

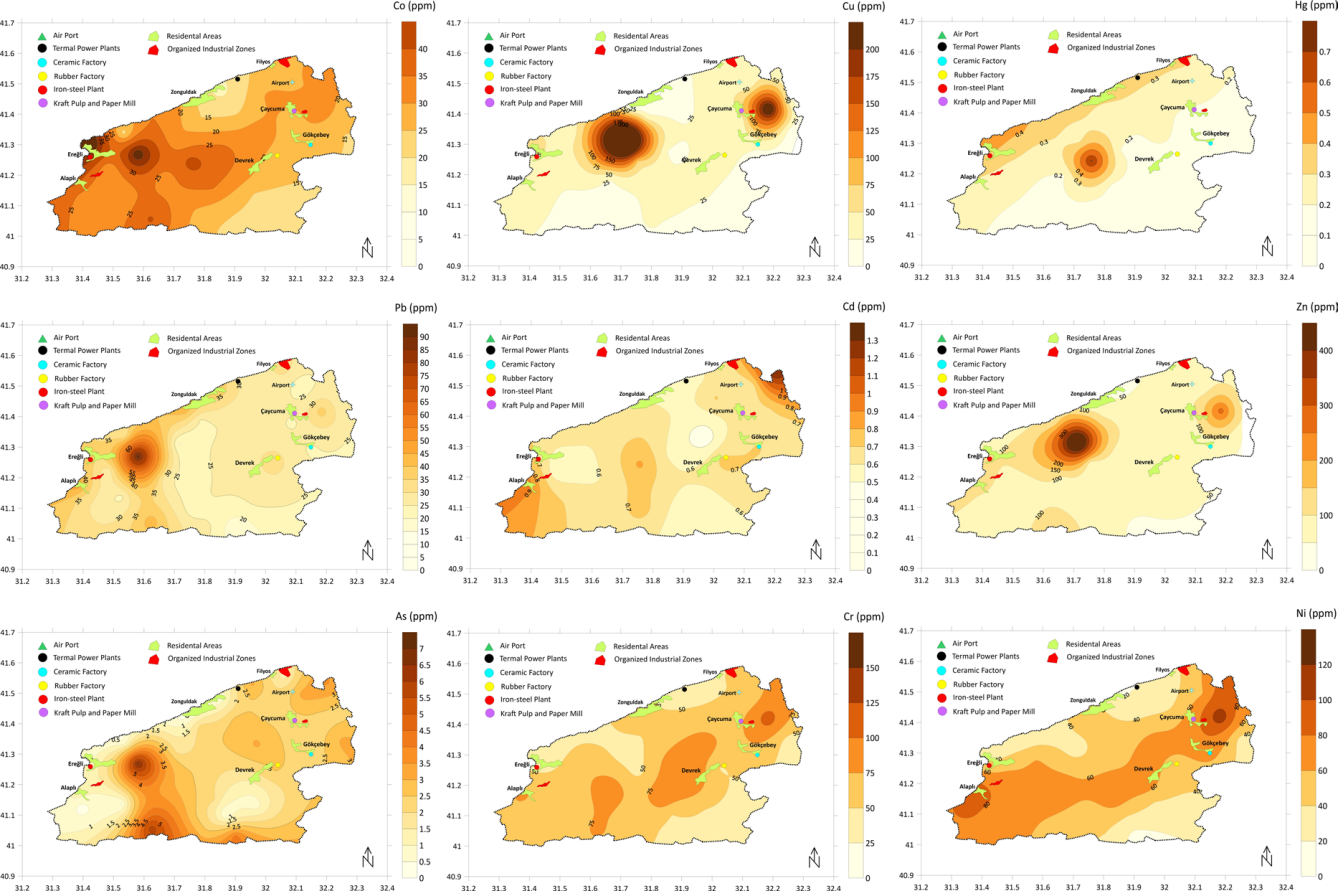
Spatial distribution of heavy metals

Spatial distributions of the heavy metal concentrations throughout the study area were presented in Fig. 5. Spatial distributions of Mn, Sn, Pb, and Cd were relatively homogeneous except for local elevations, particularly in the vicinity of Ereğli, Alaplı, and Zonguldak cities. The high Mn concentration in the western part of the region is probably related to high Fe levels because of their closely linked chemical behaviours. On the other hand, Pb and Cd concentrations in the west part of the region exceed the average values in this study and the reference values presented (Table S4) by two/three folds on some sampling points. High Cd concentrations above the world surface rock average in the Ereğli region were reported in another recent study, and it was related to anthropogenic effects (Bayrakli, 2023). Upstream areas of the Gülüç River were concentrated with Mn, Pb, and As. Especially Mn and Pb were homogenously distributed throughout the study area, except for the highlighted river basin. High As levels are affecting a larger area towards the south. Correlation and HCA results also confirm the relationship between these elements (Figs. 3 and 4). While Sn levels (2.54 ppm on average) generally are in line with the reference values (Table S4), there is a drastic increase (> 11 ppm) around Zonguldak city.

Another group of elements that had a similar distribution pattern includes Fe, Co, Cr, and Ni. Concentrations of metals in this group are generally higher in the central parts of the region, along a line extending from east to west. Concentrations increase further on the western flank of this line, particularly around the cities of Ereğli and Alaplı. Average Co and Ni concentrations of the whole study area were higher than the reference values presented (Table S4). When we focused on the Ereğli-Alaplı area, we also assessed that there are several sampling points with high values above the mean values of this study and reference values presented in Table S4. It should be noted that Cr–Ni and Fe–Co were highly correlated according to correlation analysis (Fig. 3).

Mo, Ba, and Sb, with moderate variability (CV values 0.76, 0.58, and 0.58, respectively), manifested similar distributions to a large extent. For these three elements, higher concentrations were observed, especially in the upstream areas of the Gülüç River and Alaplı River basins, in which limestone formations are dominant. Another hotspot for these three elements is Zonguldak city and its surroundings. Sr concentrations are high in the east and west parts of the study area, exceeding 300 ppm in some locations. Moreover, V concentrations exceed 125 ppm in some regions. These levels are mostly in line with world soil averages or crustal averages given in Table S4.

The other two metals with similar spatial distributions are Cu and Zn. An increase in the concentrations of both metals is observed in the area between the cities of Zonguldak, Ereğli, and Devrek, and in the east of the city of Çaycuma. At many points in these regions, Cu and Zn levels exceeded the study area average and the reference values given in Table S4. The Cu and Zn averages calculated for the study area as a whole were also higher than the reference values. The correlation and HCA results indicate a significantly strong relationship between these two metals, which should be taken into account when identifying possible sources.

When examining the spatial distribution of Hg, one of the heavy metals posing a high risk to environmental health, it is observed that concentrations remain high along the northern boundaries of the study area. While Hg levels across the study area are above reference values (Table S4), Hg values along the aforementioned line increase to levels up to 1.8 times the regional average (0.25 ppm).

Concentration levels of several heavy metals (Pb, Hg, Cd, As, Cr, Ni, Zn, V, Mn, Mo, Sr) are higher in the west–northwest part of the region, particularly in the vicinity of Ereğli and Alaplı cities. Concentration levels of Pb, Hg, Cd, Ni, Zn, V, Mn, Mo, and Sr specifically in this location are higher than crustal and average soil values given in Table S4. Especially Hg, Cd, Zn, and Ni deserve attention because their general averages were also higher than reference values. This location hosts one of the largest iron–steel industries of the country, and besides that, there is an organized industrial zone including different sectors.

### Risk assessment using enrichment/pollution indices

**Fig. 6:**
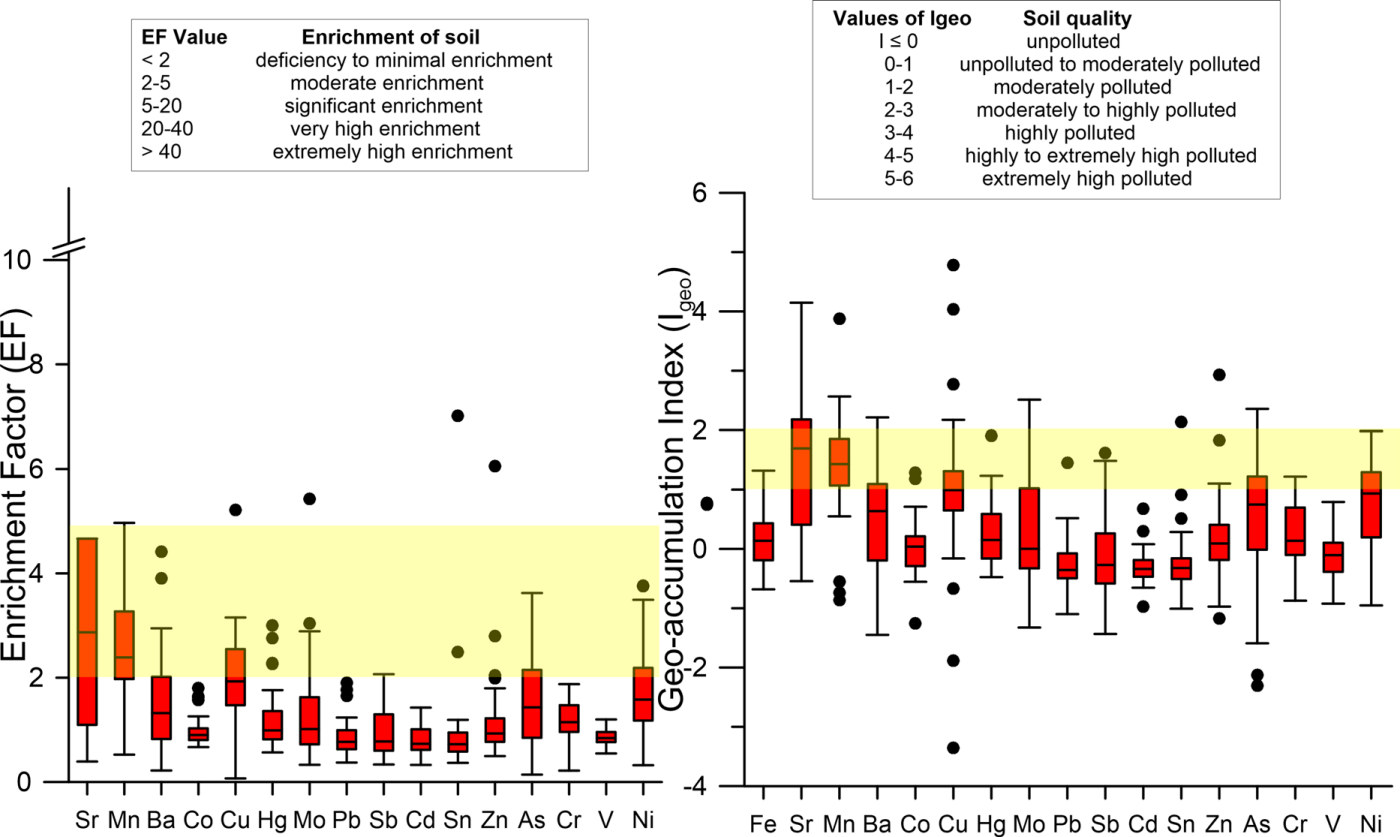
EF and Igeo index values (The areas highlighted in yellow indicate moderate enrichment/pollution)

The box plots showing EF and Igeo results of 36 sampling points across the study area were presented in Fig. 6. The results of the two indices were mostly in line. The median EF and Igeo values of Sr, Mn, and Cu fell into the moderate enrichment category. High enrichment was not present for any of the metals based on average values; however, there were several individual sampling points with high index values, especially for Ba, Hg, Mo, Zn, As, and Ni, besides the metals mentioned before.

69% of Mn EF values and 81% of Igeo values were classified as moderate enrichment, indicating a relatively homogeneous enrichment trend across the study area. In terms of spatial distribution, Mn concentrations were particularly high in the Gülüç River basin. The EF (with 1 sample showing significant enrichment and 6 samples moderate enrichment) and Igeo (4 samples moderately to highly polluted, 4 samples moderately polluted) values from this basin and the cities of Ereğli and Alaplı were significantly higher than the study area average. For example, while the study area EF average for Mn is 2.8, the average for the 7 sampling points in this region was calculated as 4.5. Thus, it can be suggested that the overall enrichment in the region is due to geogenic effects, but there are indications of anthropogenic enhancement in the specific area in question. Fifty-eight percent of Sr EF values and 61% of Igeo values were classified as at least moderately enriched. However, the averages of the 4 samples around Alaplı city and the 4 samples around Çaycuma city are 15.7 and 6.0 for EF, and 1.9 and 1.6 for Igeo, respectively. Considering that the overall averages are 5.3 and 1.6, limited anthropogenic effects may be present. 50% of the Cu EF values and 44% of the Igeo values were classified as at least moderate enrichment. These values were calculated mainly for samples from the Gülüç River basin, the cities of Ereğli and Alaplı, and the vicinity of Çaycuma city. Potential sources of Cu enrichment have been examined in detail in section "Source apportionment (PMF analysis)" (Source Apportionment).

The high index values observed for Ba, Hg, Mo, Zn, As, and Ni are mostly concentrated in specific areas. For example, most of the points classified as moderate enrichment for Ba and Mo are located in the northeast of the study area, around the cities of Zonguldak and Çaycuma. According to Hg results, the four points classified as moderate enrichment are also in the northern half of the study area, and the index averages of the 16 northern points (EF 1.5 and Igeo 0.60) are higher than the averages of the southern points (EF 0.96, Igeo-0.05). This situation also coincides with the spatial distribution results of Hg. According to Ni index values, the points classified as moderate enrichment are in the Alaplı—Gülüç basin (EF values range from 1.5 to 3.8 and Igeo values range from 1.1 to 1.7) and the eastern part of Çaycuma city (EF values range from 1.5 to 3.2 and Igeo values range from 1.0 to 2.0). Zn results also show spatial similarity with Ni, but the index values indicate weaker enrichment. Looking at the distribution of high As index values, it can be seen that they are spread across many areas. These areas are the Gülüç River Basin, the southern boundaries of the study area, the east of Zonguldak city, and the east-northeast of Çaycuma city. A total of 10 sampling points have been classified as moderate enrichment in these areas.

### Source apportionment (PMF analysis)

A comprehensive approach was used in selecting the heavy metals to be included in the PMF analysis, taking all data into account. When making the selection, it was considered that obtaining a robust model would become more difficult as the number of metals increased. Cu, Hg, Zn, As, and Ni were analyzed because the index results indicated enrichment in various regions. Furthermore, Cd, Pb, and Cr were also included in the analysis. These elements are primary environmental concerns, and although their index data did not directly show enrichment, their general or local averages were above the reference values (Table S4) or regional averages. Although CV and index values were high, Mn and Sr were not preferred due to reasons such as their concentration averages being close to or lower than crustal average values, statistical analyses generally pointing to geogenic processes, and limited anthropogenic effects.

**Fig. 7:**
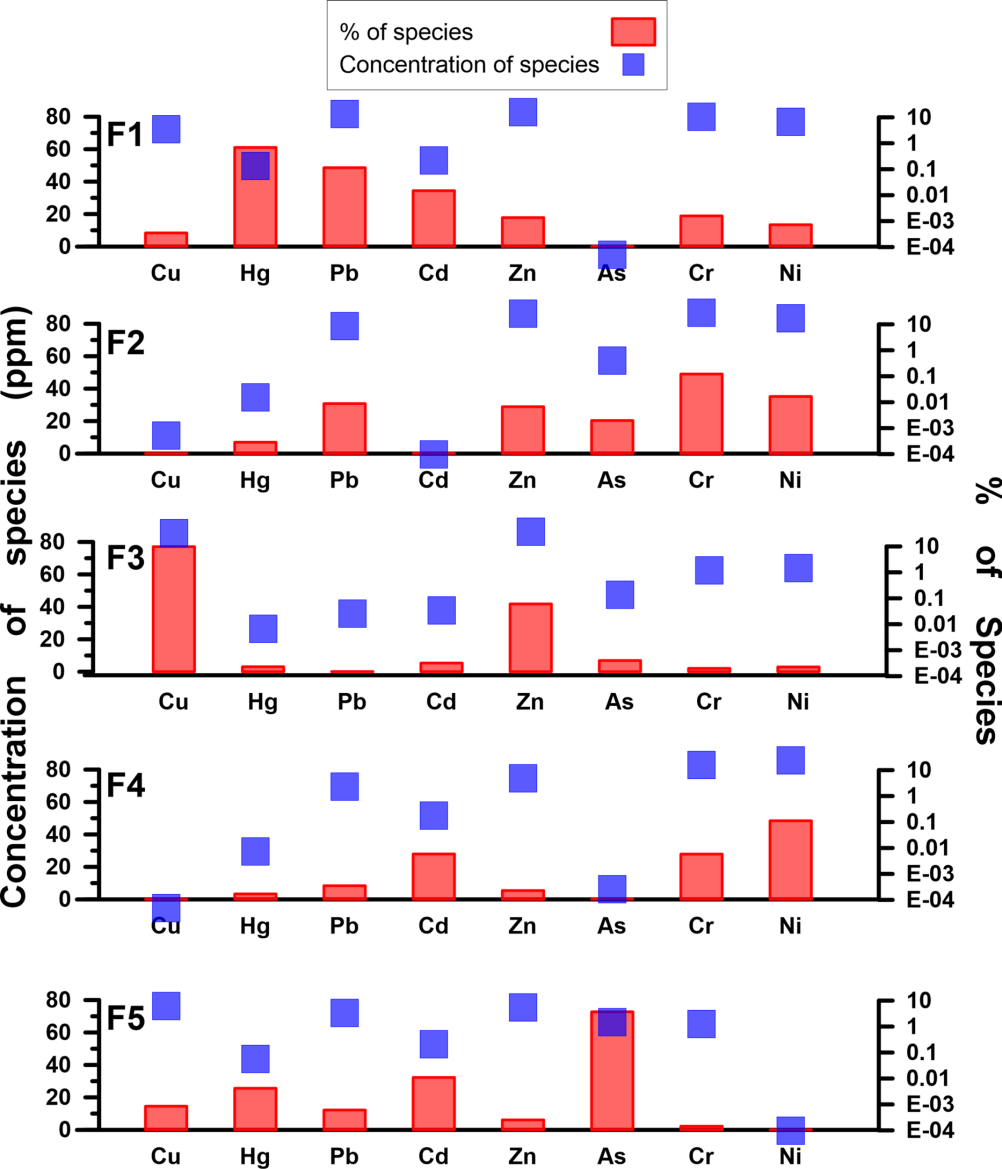
Factor profiles

**Fig. 8:**
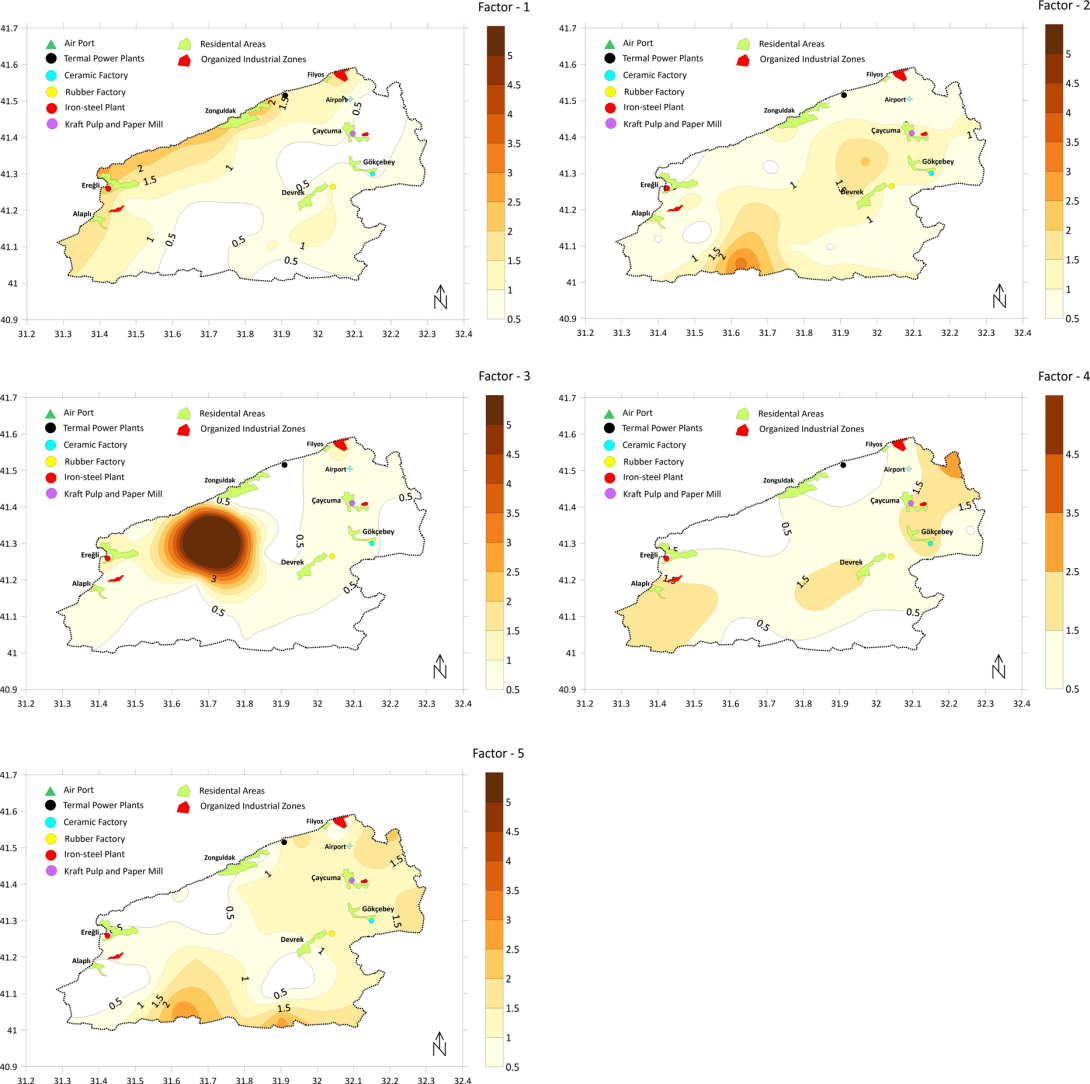
Spatial distributions of source contributions obtained from PMF analysis

As a result of multiple PMF analyses with different factor numbers, the 5-factor solution was the best to explain heavy metal concentration data in terms of both interpretability and model parameters. The 4-factor solution was not able to distinguish some of the important sources and the 6-factor solution led to decreased interpretability due to the overlapping of some source profiles. In 5-factor model, the R^2^ values of most of the heavy metals were higher than 0.90 except Cd, Cr, and Hg (0.88, 0.85, and 0.71, respectively). The smallest and most stable Q value was accepted, and almost all of the residuals were between − 3 and  + 3. These results suggest that the chosen model was acceptable. Factor profiles from the 5-factor solution and spatial distributions of source contributions obtained from PMF analysis were presented in Figs. 7 and 8.

Factor 1 is dominated by Hg (61.1%), although there are also considerable loads of Pb and Cd (48.6 and 34.4%, respectively) (Fig. 7). When examining the regions heavily affected by Factor 1, it was concluded that the distribution of Hg concentrations and pollution indices corresponded to these regions, and that the Hg enrichment detected in these areas originated from Factor 1 (Fig. 8). The CV value of Hg data (0.96) clearly indicated anthropogenic origin, while this was not the case for Pb and Cd (0.43 and 0.23, respectively) (Table 1). Although no enrichment was detected in the region based on the calculated index values for Pb, the spatial distribution of Pb concentrations, with higher concentrations along the northern and western boundaries, may be an effect of Factor 1. A similar situation applies to Cd.

According to spatial analysis results, Cd concentrations are elevated in the Ereğli–Alaplı region (Fig. 5). Coal mining and coal combustion are regarded as main sources of Hg enrichment (Fei et al., 2020; Zhang & Zhang, 2021). Especially, atmospheric deposition via wet and dry cycles is a widely suspected mechanism (Wang et al., 2024). The northern coast of the region, particularly the city of Zonguldak and its surrounding area, has historically been an area of intense coal mining activity. Coal-fired thermal power plants are still operating, and although coal has been gradually replaced by natural gas in recent years, it remains a significant source of domestic heating. The fact that enrichments are generally observed south-southwest of the thermal power plant might be an indication that wet or dry deposition following atmospheric transport is a potent mechanism in this area. In order to make more definitive comments on this subject, more intensive sampling should be carried out in the thermal power plant area, and the results should be analyzed using detailed wind data. Intense coal mining activities, coal-based residential heating, and coal-fired thermal power plants appear to be the main drivers for Factor 1.

A considerable load of Cr (49.0%) was found at Factor 2 while other contributions were from Ni, Pb, Zn and As (35.2, 30.8, 28.8 and 20.3% respectively) (Fig. 7). Correlation analysis (Fig. 3) and HCA (Fig. 4) showed that there is a close relationship between Ni and Cr (Correlation constant, Cr–Ni, r = 0.84). Ni and Cr concentrations were to a large extent similarly distributed throughout the study area (Fig. 5) and a similar pattern can be observed in Factor 2 source contributions (Fig. 8). For Ni, 31% of EF and 42% of Igeo values were above moderately enrichment level while for Cr only one value among the EF and Igeo values was above moderately enrichment level (Fig. 6). There are several studies in which Ni and Cr were reported as originating from parent material (Su et al., 2022; Wang et al., 2024; Zhang & Zhang, 2021). These results suggest that there is limited human contribution in Factor 2, and it has a geogenic origin. Furthermore, one of the previous studies focusing on Zonguldak soils supports this finding (Bayrakli, 2023).

Cu (77.1%) and Zn (41.8%) were the main components of Factor 3, which mainly comes from the region between Zonguldak–Çaycuma–Devrek cities (Fig. 7). Gülüç River basin, which includes Kızılcapınar Dam, is also a part of this area. When considering the spatial distribution of Cu and Zn concentration and index data, it is understood that these metals are also enriched in the east of Çaycuma city. The fact that this situation is not reflected in the source contribution distribution in Fig. 8 is due to samples that were not included in the PMF analysis because of outlier values. Cu and Zn were highly correlated (r = 0.95) (Fig. 3) and clustered closely according to HCA (Fig. 4), which indicated a shared source. CV values of both of the metals are extremely high (1.52 and 0.90 for Cu and Zn, respectively) (Table 1), and this also hints that the source might be due to human activities. 50% of both EF and Igeo values for Cu were calculated above the moderate level enrichment threshold, while this value was 8.3% for Zn. It was suspected that several sampling points classified as moderate level enrichment were in or close to areas highly affected by Factor 3. Livestock farming is an important source of income in Zonguldak Province and especially in Çaycuma city and surroundings (Keskin Citiroglu et al., 2017). Livestock farming activities are also carried out in the Gülüç River basin (Kızılcapınar Reservoir Basin), located in the source area of Factor 3. Indeed, it has been determined that pollution from livestock farming activities in the Western Black Sea Basin is a significant problem in both groundwater and surface water. Furthermore, in the risk assessment of the basin as a whole, it has been determined that livestock farming poses a “Very High Risk” to 26 surface water bodies (Ministry of Environment, Urbanization and Climate Change of the Republic of Turkey, 2025). In this context, the Kızılcapınar Dam Protection Plan contains specific provisions requiring the construction of leak-proof manure pits for the management and disposal of animal waste in the basin and the use of manure under appropriate conditions or its removal from the basin (Ministry of Agriculture & Forestry of the Republic of Turkey, 2024). Therefore, since Cu and Zn are accepted as animal manure indicators, these metals also have the potential to accumulate in the Kızılcapınar Dam basin. The inefficient metabolic uptake of zinc and copper from feed additives of livestock leads to their concentration in animal feces, and they are considered indicators of livestock manure (Su et al., 2022). They also originate from other farming activities such as pesticide and fertilizer usage (Wang et al., 2024; Zhang & Zhang, 2021). Therefore, Factor 3 might be mainly livestock farming-related agricultural activities.

The heavy metals with the highest weight in Factor 4 were Ni, Cr, and Cd (48.4, 27.8, and 28.0, respectively) (Fig. 7). Different types of industrial facilities are found in the regions where Factor 4 is predominant. Organized industrial zones, iron and steel, ceramics, and paper industries, can be considered as sources that are effective in these regions. Main pollutants originating from iron–iron-steel industry are documented as As, Cr, Pb, Mn, Mo, Ni, Se, Sb, W, V, Zn (Alloway, 2013). Therefore, it is highly probable that the iron-steel industry has a contribution to this factor because other metals in this factor besides Ni and Cr are Pb, Zn, and Hg (8.4, 5.4, and 3.3% respectively). On the other hand, the similarity of the spatial distribution of this factor to Factor 2 indicates that geogenic sources may also be considered. It has been reported that the use of phosphorus fertilizers, particularly in agricultural activities such as hazelnut production in the cities of Ereğli and Alaplı, may have contributed to the increase in Cd levels (Bayrakli, 2023). The spatial distribution of these areas also indicates that agricultural effects are present in Factor 4. Ultimately, it can be said that Factor 4 predominantly represents mixed industrial sources.

Factor 5 has major contributions from As, Cd, and Hg (72.7, 32.3, and 25.6%, respectively) (Fig. 7). In the spatial distribution map shown in Fig. 8, the areas where Factor 5 is dominant coincide with the concentration and index data belonging to As. In the southern part of the study area, which is located among these areas, there are no intensive industrial or domestic sources, and Factor 2, classified as geogenic, is also dominant in this region. Therefore, parent material Factor 5 may also be effective. The other regions are the south and east of Zonguldak city and the east-northeast regions of Çaycuma city. Indices for As on the sampling point close to the thermal power plant are 2.44 for EF and 1.24 for Igeo (moderate enrichment), which supports the impact of the thermal power plant. Coal ash might contain elevated levels of As depending on the coal composition and ash fraction (Wenzel, 2013). One of the other possible sources might be agricultural activities. As mentioned in the section on Factor 3, livestock farming is particularly widespread in the large area surrounding the city of Çaycuma. Therefore, it can be said that Factor 5 is a resource composed of multiple sources.

The main sources of heavy metal enrichment in the study area are from five potential sources based on the PMF analysis: Factor 1 is coal mining and coal–based emission sources, Factor 2 is soil parent material, Factor 3 is agricultural pollution sources, Factor 4 is industrial sources, and Factor 5 is mixed sources.

The severity and distribution of soil heavy metal contamination in Zonguldak province show both similarities and region-specific differences when compared to other industrial areas with coal mining on a global scale. For example, in the Appalachian coal basin (USA), Fe, Mn, and SO_4_ enrichment is caused by acid mine drainage, generally resulting from the oxidative dissolution of pyrite, and metals such as Cr, Cu, and Ni come to the fore (Chowdhury & Singer, 2024; Siegel et al., 2022). In this study, the fact that Co, Cu, Hg, Cd, Zn, and Ni exceeding global averages, indicates that similar mechanisms may be at work, but region-specific factors also contribute. Globally, the metals with the highest enrichment detected in regions with intensive coal mining activities are reported to be As, Bi, Hg, Sb, and Se (Alekseenko et al., 2025). In Zonguldak, Hg contamination is directly linked to coal mining and combustion (Factor 1), while it is noteworthy that As levels (average 2.23 mg/kg) are well below the global coal mine soil average (15.9 mg/kg) (Alekseenko et al., 2025). As the PMF results also indicate, the most important aspect distinguishing Zonguldak from other regions is its multi-layered enrichment profile. These findings suggest that metal accumulation in Zonguldak is not solely mining-based but rather a synergistic result of local geology and multi-sector industrial emissions.

### Ecological risk assessment

**Fig. 9:**
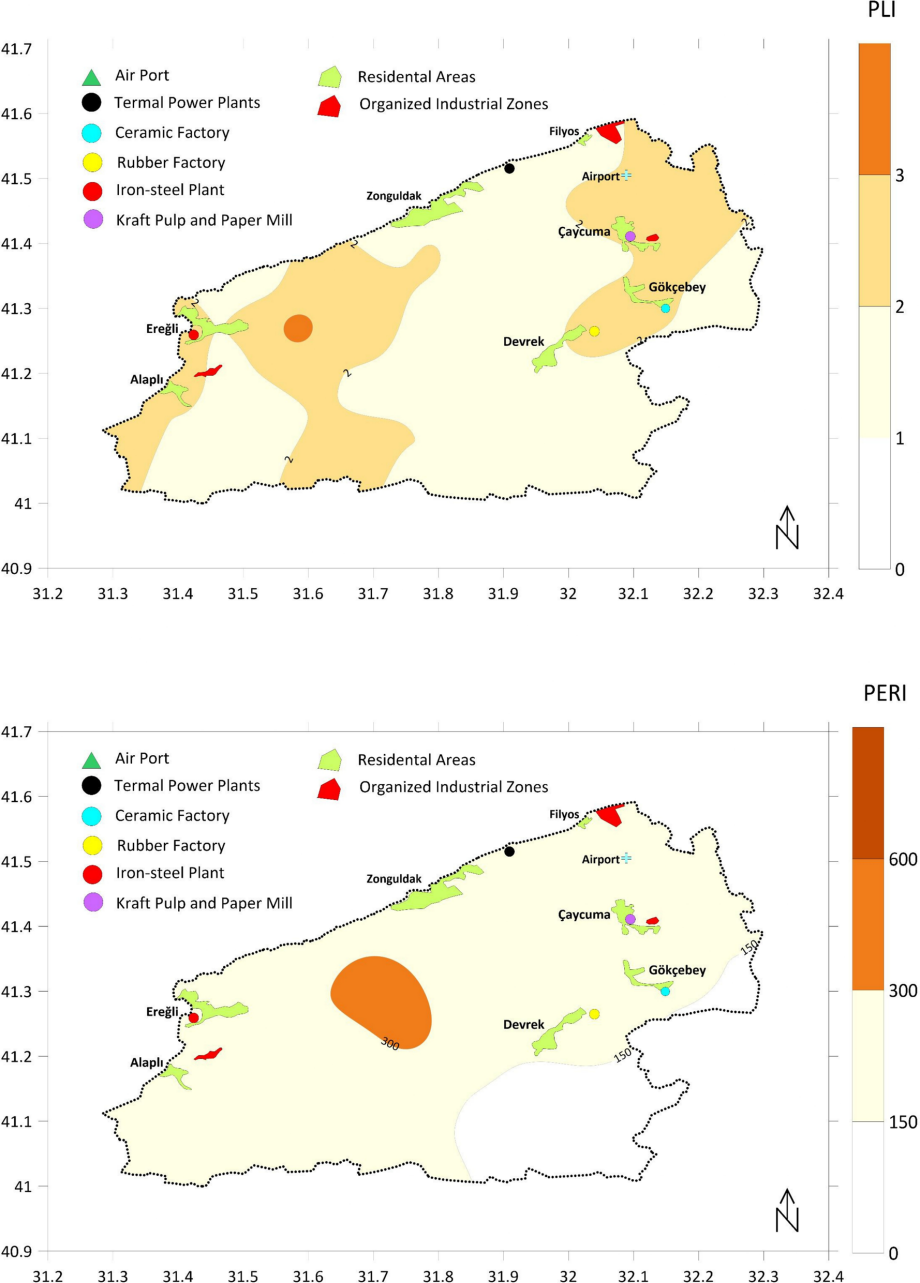
Spatial distributions of PLI and PERI indices

Figure 9 shows the results of PLI and PERI indices. PLI classification considers values lower than 1 as “denotes perfection” and greater than 1 as “deterioration of soil quality” and does not implement further labeling. According to the spatial distribution of PLI index results in Fig. 9, it is evident that there is some degree of deterioration in soil quality in Zonguldak soils regardless of location. Index values are apparently higher on the west part, particularly around Ereğli city, Alaplı city, and Gülüç River basin, and on the east, particularly around Çaycuma city, Filyos River basin, Gökçebey city, and Devrek city. PLI values are mostly above 2 in these regions, suggesting a much more severe deterioration in soil quality. A similar pattern was also observed in PERI results (Fig. 9). Most of the study area was under the category of “moderate ecological risk”, including the areas highlighted in PLI results. The only location with a “high potential ecological risk” value was the upstream part of the Gülüç River basin, which also hosts a main domestic water source, the Kızılcapınar dam.

**Fig. 10:**
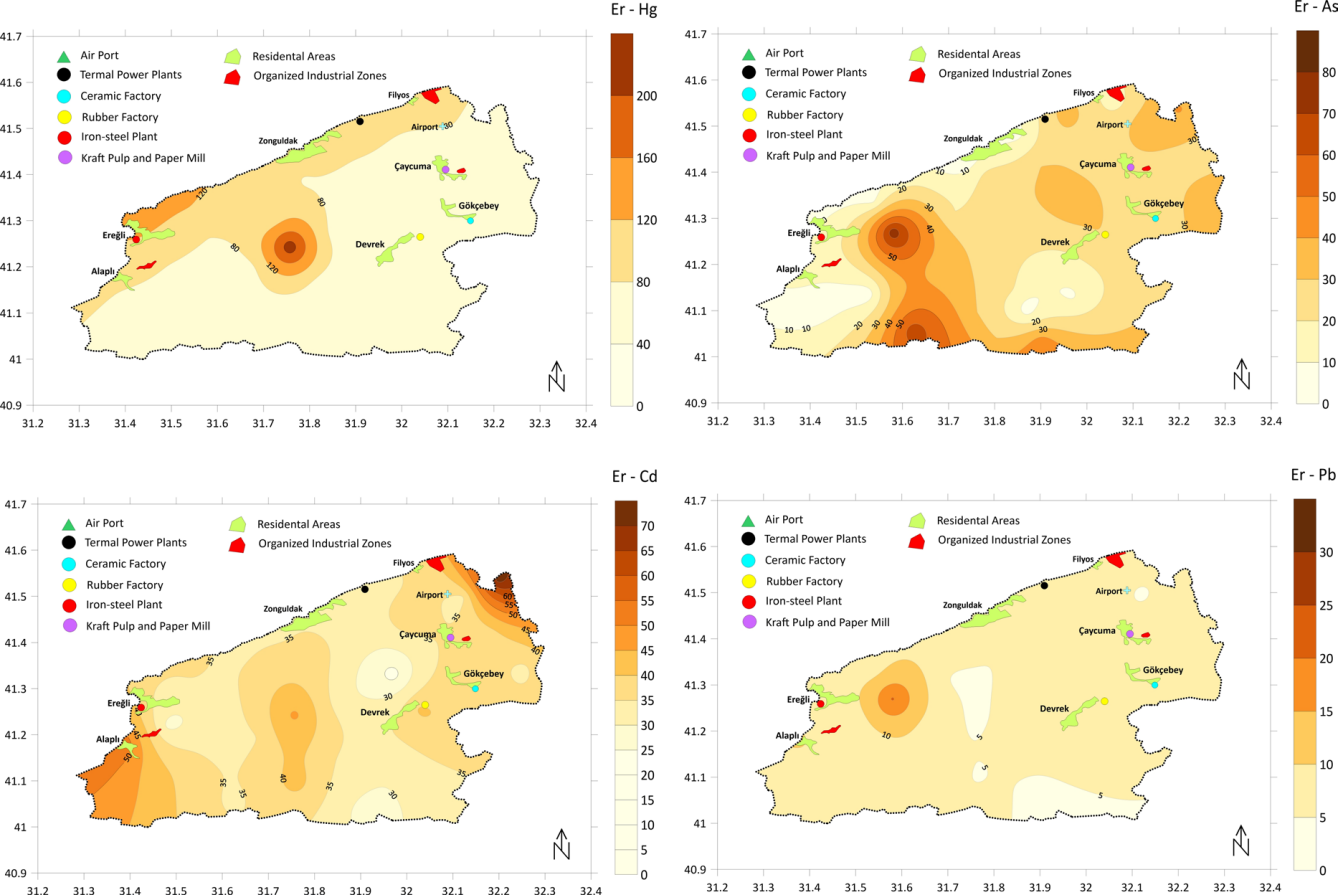
Spatial distribution of Er index values of selected heavy metals

PERI index is the summation of individual Er index values of several heavy metals, and these Er values can be interpreted individually to draw a conclusion about the potential ecological risk posed by Hg, Cd, Co, Cu, Pb, Ni, Cr, As, and Zn. Heavy metals for which Er values can be calculated were ranked as follows, from the highest average Er to the lowest: Hg > Cd > As > Cu > Ni > Co > Pb > Cr > Zn. Figure 10 shows the spatial distribution of Er values for Hg, Cd, and As metals, which reached the highest ecological risk coefficients. Based on the Er classification (40 < Er < 80 Moderate ecological risk) and average Er values, Hg level poses moderate ecological risk (average Er 76.6) while Cd and As are the closest heavy metals to this category (average Er values are 37.3 and 25.3, respectively). Spatial distribution of Er values of these metals is presented in Fig. 10. Along the northern boundary of the study area, Hg enrichment has been observed to exceed the “considerable ecological risk” threshold. This situation continues inland along the Gülüç River basin. Although As values are also high in this region, they do not exceed the “moderate ecological risk” level. Looking at the Cd results, Er values are elevated in the cities of Ereğli-Alaplı and the eastern part of Çaycuma, but these increases remain at the “moderate ecological risk” level.

In a region such as the Zonguldak province, with its history of mining and heavy industry, it is inevitable that there will be ethical implications of the pressure exerted on environmental health. In this context, the risks posed by pollutants such as heavy metals on soil and water resources must be addressed from an environmental ethics perspective. This situation is a fundamental reflection of the concept of environmental justice, which questions the disproportionate distribution of environmental burdens and risks among different segments of society. The local population, predominantly farmers and workers who depend on the region’s water resources and agricultural activities for their basic drinking water and food needs, can be considered vulnerable groups against the effects of potential heavy metal pollution. The limited ability of these communities, with their low socioeconomic resources, to leave contaminated areas or access alternative clean food sources increases their exposure risk and shifts the issue from a technical problem to an ethical responsibility. Pollution caused by industrial emissions and mining waste has historically been linked to serious health problems among the local population, such as pneumoconiosis, goiter, and cancer (Turer et al., 2008). The careful identification of these and similar risks is important in order to eliminate the threats posed by environmental pollution not only to ecosystems but also to the right to life of future generations. Within an ethical management framework, protecting this vulnerable population should be as much of a priority as detecting pollution. To effectively manage these risks, a strong stakeholder engagement strategy must be implemented among scientists, government agencies, established industry stakeholders, investors, and local communities. This collaboration should encompass not only technical pollution analyses but also integrated protection plans that take into account the socio-economic needs of the local community.

## Conclusion

This research presents an integrated assessment—combining statistical evaluation, spatial mapping, and multiple pollution indices (I_geo_, EF, PERI, PLI, Er) with Positive Matrix Factorization (PMF)—to evaluate heavy metal (HM) contamination and identify potential sources in the industrialized coal-mining region of Zonguldak, Turkey. The spatial analysis indicated certain pollution hotspots near Ereğli, Alaplı, Zonguldak, and the Gülüç River basin, suggesting that metal enrichment is closely linked to specific localized activities. PMF analysis attributed these enrichments to five potential sources: coal mining and coal-based emissions, geogenic origin, industrial activities, agricultural sources, and mixed anthropogenic inputs. The ecological risk assessment suggested that soil quality deterioration is present across the province (PLI > 1), with Mercury (Hg) emerging as a notable individual risk factor based on its average Er value.

As this study is exploratory in nature, the findings provide a scientific basis for preliminary environmental management in the region. The results indicate that continuous monitoring may be necessary at points where industrial infrastructure intersects with urban areas. The most critical finding is that Hg has been identified as the primary individual ecological risk factor, indicating an urgent need for targeted monitoring strategies. To develop a regional overview, future research could focus on more intensive sampling strategies at the identified focal points and include human health risk assessments to evaluate the long-term impacts on local communities more comprehensively. Ultimately, this integrated framework offers a useful starting point for transitioning to source-specific monitoring and remediation efforts in coal-dependent industrial areas.

## Supplementary Information

Below is the link to the electronic supplementary material.

Supplementary file1 (DOCX 70 KB)

## Data Availability

The relevant data from this study are available from the corresponding author upon request.
