# Supplementary material for: Assessment of potential contamination, ecological risk, spatial analysis, and source apportionment of soil heavy metals in a highly industrialized coal–mining region
Source: Environ Geochem Health. 2026 Mar 2;48(5):209. doi: 10.1007/s10653-026-03099-7 (PMC12953411; doi:10.1007/s10653-026-03099-7)
Supplement: Supplementary file 1 — Supplementary file1 (DOCX 70 KB) [file 10653_2026_3099_MOESM1_ESM.docx]

**Assessment of potential contamination, ecological risk, spatial analysis, and source apportionment of soil heavy metals in a highly industrialized coal – mining region**

Ahmet Altin^a^, Bekir Fatih Kahraman^a,*^, Sinem Çolak^b^, Koray Alper^b^, Süreyya Altin^a^, Ferruh Niyazi Ayoğlu^c^

^a^Department of Environmental Engineering, Zonguldak Bülent Ecevit University, , Zonguldak, 67100, Turkey

^b^Department of Chemistry and Chemical Processing, Caycuma Vocational School of Food and Agriculture, Zonguldak Bülent Ecevit University, Caycuma, Zonguldak, 67900, Turkey

^c^Department of Public Health, Faculty of Medicine, Bulent Ecevit University, Zonguldak, 67100, Turkey

Table S1. Coordinates of the Zonguldak province-wide sampling points

| **Sampling Point** | **Latitude** | **Longitude** | **Sampling Point** | **Latitude** | **Longitude** |
| --- | --- | --- | --- | --- | --- |
| Z1 | 41.1755° | 31.3818° | Z19 | 41.3883° | 31.7880° |
| Z2 | 41.1262° | 31.3953° | Z20 | 41.2763° | 31.8588° |
| Z3 | 41.3015° | 31.4248° | Z21 | 41.2065° | 31.9377° |
| Z4 | 41.2206° | 31.4831° | Z22 | 41.1317° | 31.9686° |
| Z5 | 41.1363° | 31.5257° | Z23 | 41.4829° | 31.8501° |
| Z6 | 41.0560° | 31.6234° | Z24 | 41.4101° | 31.9022° |
| Z7 | 41.3390° | 31.5346° | Z25 | 41.3314° | 31.9708° |
| Z8 | 41.2698° | 31.5797° | Z26 | 41.2545° | 32.0290° |
| Z9 | 41.1923° | 31.6670° | Z27 | 41.5308° | 31.9561° |
| Z10 | 41.0964° | 31.7614° | Z28 | 41.4591° | 32.0072° |
| Z11 | 41.0392° | 31.8037° | Z29 | 41.3786° | 32.0910° |
| Z12 | 41.3814° | 31.6310° | Z30 | 41.2959° | 32.1483° |
| Z13 | 41.3127° | 31.7127° | Z31 | 41.5569° | 32.0602° |
| Z14 | 41.2426° | 31.7570° | Z32 | 41.4964° | 32.1198° |
| Z15 | 41.1510° | 31.8305° | Z33 | 41.4166° | 32.1838° |
| Z16 | 41.0996° | 31.8694° | Z34 | 41.3428° | 32.2527° |
| Z17 | 41.0305° | 31.9013° | Z35 | 41.5360° | 32.1987° |
| Z18 | 41.4239° | 31.7316° | Z36 | 41.4458° | 32.2536° |

Table S2. Temperature program of the microwave digestion procedure

| **Parameter** | **Step 1** | **Step 2** | **Step 3** | **Step 4** | **Step 5** |
| --- | --- | --- | --- | --- | --- |
| **Temperature (°C)** | 150 | 220 | 200 | 150 | 100 |
| **Time (min)** | 10 | 15 | 5 | 5 | 1 |
| **Ramp (min)** | 2 | 2 | 2 | 2 | 2 |

Table S3. The detection limit (LOD), limit of quantification (LOQ) and recovery percentages for 17 elements.

| **Elements** | **LOD (mg.kg**^−1^**)** | **LOQ (mg.kg**^−1^**)** | **Recovery (%)** | **Elements** | **LOD (mg/kg)** | **LOQ (mg/kg)** | **Recovery (%)** |
| --- | --- | --- | --- | --- | --- | --- | --- |
| Fe | 0.017 | 0.053 | 110.4 | Sb | 0.003 | 0.008 | 83.7 |
| Sr | 0.014 | 0.042 | 100.4 | Cd | 0.003 | 0.009 | 89.1 |
| Mn | 0.016 | 0.049 | 89.9 | Sn | 0.004 | 0.011 | 97.0 |
| Ba | 0.012 | 0.037 | 82.6 | Zn | 0.007 | 0.021 | 62.3 |
| Co | 0.003 | 0.009 | 78.8 | As | 0.004 | 0.011 | 79.8 |
| Cu | 0.005 | 0.014 | 73.8 | Cr | 0.007 | 0.022 | 90.5 |
| Hg | 0.006 | 0.019 | 73.7 | V | 0.003 | 0.009 | 100.4 |
| Mo | 0.003 | 0.008 | 83.6 | Ni | 0.001 | 0.003 | 79.5 |
| Pb | 0.005 | 0.015 | 88.0 |  |  |  |  |

Table S4. Mean background concentrations of elements in continental crust, surface soils and soils of different countries and cities with industrial activities (mg/kg).

|  | **Fe** | **Sr** | **Mn** | **Ba** | **Co** | **Cu** | **Hg** | **Mo** | **Pb** | **Sb** | **Cd** | **Sn** | **Zn** | **As** | **Cr** | **V** | **Ni** |
| --- | --- | --- | --- | --- | --- | --- | --- | --- | --- | --- | --- | --- | --- | --- | --- | --- | --- |
| **This study** | 41153 | 131.50 | 999.31 | 286.67 | 21.55 | 51.14 | 0.25 | 1.07 | 30.68 | 0.38 | 0.67 | 2.54 | 101.6 | 2.23 | 61.38 | 121.22 | 53.66 |
| **World-soil average** (Kabata-Pendias, 2011) |  | 175 | 488 | 460 | 11.3 | 38.9 | 0.07 | 1.1 | 27 | 0.67 | 0.41 | 2.5 | 70 | 6.83 | 59.5 | 129 | 29 |
| **Top soils of Europe** (Kabata-Pendias, 2011) |  | 130 | 524 | 400 | 10.4 | 17.3 | 0.06 | 0.94 | 32 | 1.04 | 0.28 | 4.5 | 68.1 | 11.6 | 94.8 | 68 | 37 |
| **Upper Continental Crust** (Wedepohl, 1995) | 30890 | 316 | 527 | 668 | 11.6 | 14.3 | 0.056 | 1.4 | 17 | 0.31 | 0.106 | 2.5 | 52 | 2 | 35 | 53 | 18.6 |
| **Sandstones** (Turekian & Wedepohl, 1961) | 9800 | 20 | X0. | X0. | 0.3 | X. | 0.03 | 0.2 | 7 | 0.0X | 0.0X | 0.X | 16 | 1 | 35 | 20 | 2 |
| **Carbonates** (Turekian & Wedepohl, 1961) | 3800 | 610 | 1100 | 0. | 0.1 | 4 | 0.04 | 0.4 | 9 | 0.2 | 0.035 | 0.X | 20 | 1 | 11 | 20 | X0. |
| **Deep Sea Sediments** (Turekian & Wedepohl, 1961) | 9000 | 2000 | 1000 | 190 | 7 | 30 | 0.0X | 3 | 9 | 0.15 | 0.0X | 0.X | 35 | 1 | 11 | 20 | 30 |
| **Granitic rocks** (Turekian & Wedepohl, 1961) | 29600 | 440 | 540 | 420 | 7 | 30 | 0.08 | 1 | 15 | 0.2 | 0.13 | 1.5 | 60 | 1.9 | 22 | 88 | 15 |
| **Agricultural district, Zonguldak, Turkey** (Bayrakli, 2023) | 25033 |  | 890.3 |  | 8.49 | 29.65 |  |  | 8.15 |  | 0.62 |  | 61.98 |  | 47.42 |  | 42.07 |
| **Industrial area, Gebze,**  **Turkey** (Yaylalı-Abanuz, 2011) |  |  | 1824 |  |  | 95.88 | 0.08 |  | 246 |  | 0.1 |  | 632 | 9.53 | 118 |  |  |

Table S4. Mean background concentrations of elements in continental crust, surface soils and soils of different countries and cities with industrial activities (mg/kg) (continued)

|  | **Fe** | **Sr** | **Mn** | **Ba** | **Co** | **Cu** | **Hg** | **Mo** | **Pb** | **Sb** | **Cd** | **Sn** | **Zn** | **As** | **Cr** | **V** | **Ni** |
| --- | --- | --- | --- | --- | --- | --- | --- | --- | --- | --- | --- | --- | --- | --- | --- | --- | --- |
| **This study** | 41153 | 131.50 | 999.31 | 286.67 | 21.55 | 51.14 | 0.25 | 1.07 | 30.68 | 0.38 | 0.67 | 2.54 | 101.6 | 2.23 | 61.38 | 121.22 | 53.66 |
| **Agricultural district , Eskişehir, Turkey** (Taşpınar et al., 2022) |  |  | 664.91 |  | 21.99 | 24.24 |  |  | 16.46 |  |  |  | 52.33 |  | 149.73 |  | 191.76 |
| **Coal**  **mining area, Anhui, China** (Zhu et al., 2024) |  |  |  |  |  | 31.06 |  |  | 26.57 |  | 0.27 |  | 73.09 | 13.56 | 75.08 |  | 40.02 |
| **Coal**  **mining area, LianYuan,**  **China** (Liang et al., 2017) |  |  | 552.5 |  |  | 33.26 | 178.2 | 1.63 | 37.82 | 3.79 | 0.59 |  | 107.2 | 14.96 | 93.03 | 113.6 |  |
| **Steelworks site, Cracow, Poland** (Lenart & Wolny-Koładka, 2013) | 45698 |  | 3728 |  |  | 141.86 |  |  | 213 |  | 5.31 |  | 2497 |  | 200.64 |  | 21.65 |
| **Coal mining area, Sao Pedro da Cova, Portugal** (Santos et al., 2023) |  |  |  |  | 7.23 | 50.18 |  | 2.13 | 50.22 | 6.29 | 0.11 |  | 96.97 | 22.55 | 74.10 |  | 24.29 |
| **Industrial area, Langreo, Spain** (Boente et al., 2018) |  |  |  | 107.9 | 10 | 39 | 0.4 | 1.0 | 91.6 | 2.5 | 0.6 |  | 136.2 | 21.8 |  | 27.9 | 18.3 |
| **Industrial city, Asturias, Spain** (Ordóñez et al., 2003) |  | 33 | 690 | 189 | 8.91 | 62.5 | 0.57 | 1.28 | 149 | 4.6 | 2.16 |  | 376 | 20.9 | 23.9 | 34.1 | 16.7 |

^*^The symbol X denotes order of magnitude estimates

References

Bayrakli, B. (2023). Evaluating heavy metal pollution risks and enzyme activity in soils with intensive hazelnut cultivation under humid ecological conditions. *Environmental Monitoring and Assessment*, *195*(2), 331. https://doi.org/10.1007/s10661-023-10934-2

Boente, C., Albuquerque, M. T. D., Fernández-Braña, A., Gerassis, S., Sierra, C., & Gallego, J. R. (2018). Combining raw and compositional data to determine the spatial patterns of Potentially Toxic Elements in soils. *Science of The Total Environment*, *631–632*, 1117–1126. https://doi.org/https://doi.org/10.1016/j.scitotenv.2018.03.048

Kabata-Pendias, A. (2011). *Trace Elements in Soils and Plants* (4th ed.). CRC Press.

Lenart, A., & Wolny-Koładka, K. (2013). The effect of heavy metal concentration and soil ph on the abundance of selected microbial groups within arcelormittal poland steelworks in cracow. *Bulletin of Environmental Contamination and Toxicology*, *90*(1), 85–90. https://doi.org/10.1007/s00128-012-0869-3

Liang, J., Feng, C., Zeng, G., Gao, X., Zhong, M., Li, X., Li, X., He, X., & Fang, Y. (2017). Spatial distribution and source identification of heavy metals in surface soils in a typical coal mine city, Lianyuan, China. *Environmental Pollution*, *225*, 681–690. https://doi.org/https://doi.org/10.1016/j.envpol.2017.03.057

Ordóñez, A., Loredo, J., De Miguel, E., & Charlesworth, S. (2003). Distribution of Heavy Metals in the Street Dusts and Soils of an Industrial City in Northern Spain. *Archives of Environmental Contamination and Toxicology*, *44*(2), 160–170. https://doi.org/10.1007/s00244-002-2005-6

Santos, P., Ribeiro, J., Espinha Marques, J., & Flores, D. (2023). Environmental and Health Risk Assessment of Soil Adjacent to a Self-Burning Waste Pile from an Abandoned Coal Mine in Northern Portugal. *Environments*, *10*(3), 53. https://doi.org/10.3390/environments10030053

Taşpınar, K., Ateş, Ö., Özge Pınar, M., Yalçın, G., Kızılaslan, F., & Fidantemiz, Y. F. (2022). Soil contamination assessment and potential sources of heavy metals of alpu plain Eskişehir Turkey. *International Journal of Environmental Health Research*, *32*(6), 1282–1290. https://doi.org/10.1080/09603123.2021.1876218

Turekian, K. K., & Wedepohl, K. H. (1961). Distribution of the Elements in Some Major Units of the Earth’s Crust. *Geological Society of America Bulletin*, *72*(2), 175–192.

Wedepohl, K. H. (1995). The composition of the continental crust. *Geochimica et Cosmochimica Acta*, *59*(7), 1217–1232.

Yaylalı-Abanuz, G. (2011). Heavy metal contamination of surface soil around Gebze industrial area, Turkey. *Microchemical Journal*, *99*(1), 82–92. https://doi.org/https://doi.org/10.1016/j.microc.2011.04.004

Zhu, Y., An, Y., Li, X., Cheng, L., & Lv, S. (2024). Geochemical characteristics and health risks of heavy metals in agricultural soils and crops from a coal mining area in Anhui province, China. *Environmental Research*, *241*, 117670. https://doi.org/https://doi.org/10.1016/j.envres.2023.117670
